# Advances in Cellulose-Based Hydrogels for Biomedical Engineering: A Review Summary

**DOI:** 10.3390/gels8060364

**Published:** 2022-06-08

**Authors:** Pengfei Zou, Jiaxin Yao, Ya-Nan Cui, Te Zhao, Junwei Che, Meiyan Yang, Zhiping Li, Chunsheng Gao

**Affiliations:** 1 State Key Laboratory of Toxicology and Medical Countermeasures, Beijing Institute of Pharmacology and Toxicology, Beijing 100850, China; wsygfxj@163.com (P.Z.); yaojiaxin0719@126.com (J.Y.); yanancui518@163.com (Y.-N.C.); zt12080923352021@163.com (T.Z.); chejunwei1019@163.com (J.C.); ymyzi@163.com (M.Y.); 2 School of Chemical and Pharmaceutical Engineering, Hebei University of Science and Technology, Shijiazhuang 050018, China; 3 School of Pharmaceutical Sciences, Shandong First Medical University & Shandong Academy of Medical Sciences, Taian 271016, China

**Keywords:** cellulose, hydrogels, biomedical engineering, application

## Abstract

In recent years, hydrogel-based research in biomedical engineering has attracted more attention. Cellulose-based hydrogels have become a research hotspot in the field of functional materials because of their outstanding characteristics such as excellent flexibility, stimulus-response, biocompatibility, and degradability. In addition, cellulose-based hydrogel materials exhibit excellent mechanical properties and designable functions through different preparation methods and structure designs, demonstrating huge development potential. In this review, we have systematically summarized sources and types of cellulose and the formation mechanism of the hydrogel. We have reviewed and discussed the recent progress in the development of cellulose-based hydrogels and introduced their applications such as ionic conduction, thermal insulation, and drug delivery. Also, we analyzed and highlighted the trends and opportunities for the further development of cellulose-based hydrogels as emerging materials in the future.

## 1. Introduction

Growing concerns about environmental issues and the increasing demand for environmentally friendly materials have forced researchers around the world to explore naturally occurring biopolymer or biomimetic materials for their potential applications in various fields [1,2]. Hydrogels are ductile and extremely porous polymers with a three-dimensional network structure, which was first produced by Wicherle and Lim in 1960 [3]. Over time, the research of hydrogels has developed for biomedical applications [4], including wound dressings [5,6], anti-tumor immunotherapy [7,8], anti-central nervous system disorders [9], tissue-engineering [10,11,12,13,14], smart drug-delivery systems [7,15,16], and contraception [15], due to their good biocompatibility, excellent physical and mechanical properties, and long-term implant stability.

So far, hydrogels are divided into physical hydrogels and chemical hydrogels according to the different cross-linking modes [16]. Physical hydrogels are formed by physical forces, such as hydrophobic aggregation, π-π stacking, hydrogen bonding, and electrostatic interaction, which are non-permanent and converted into a solution by heating or other external stimulation. On the contrary, chemical hydrogels formed by chemical cross-linking are permanent and irreversible. In addition, hydrogels are also divided into traditional hydrogels and functional/smart hydrogels according to their response to environmental stimuli [17]. Traditional hydrogels are not sensitive to environmental changes, while functional/smart stimuli-responsive nanocomposite hydrogels [18] produce corresponding changes in physical structure and chemical properties to small changes in the external environment (such as temperature, pH, light, magnetism, etc.) [19,20]. The outstanding feature of these hydrogels is that the swelling behavior changes significantly in response to the environment. They are used as actuators [21], sensors [22], plantable and biodegradable ion batteries [23], thermally insulating materials [24], for tissue transformation [25,26], in controlled-release switches [27,28] or in precise topical administration regimens [29] and programmable and bioinstructive materials systems [30], etc. Therefore, functional/smart stimuli-responsive hydrogels have been one of the most interesting topics for scientific researchers in recent years.

On the other hand, hydrogels can also be divided into synthetic polymer hydrogels and natural polymer hydrogels according to the different synthetic raw materials [31]. The natural polymer has attracted more attention due to its biocompatibility, abundant source, low price and good biomedical application prospects. For instance, cellulose, alginate, chitosan, pectin, and starch are the most important biopolymers used for the fabrication of biopolymer hydrogels. Amongst these natural polymers, research focusing on cellulose-based hydrogels has gained significant attention because of their low cost, strong processability, renewability, biocompatibility, biodegradability and environmental friendliness [32]. However, the poor strength of these natural hydrogels has further limited their application. To address this challenge, synthetic hydrogels and hybrid hydrogels are favored by researchers because of their tunable physical and chemical properties, which include super-adhesion [33], strong toughness, fatigue resistance, self-reinforcement [34], and self-healing [35,36,37,38]. In addition, strategies for the design and functionality of aerogels [39,40] and nanofiber-based hydrogels [41] were also considered for intensive attention.

**Figure 1:**
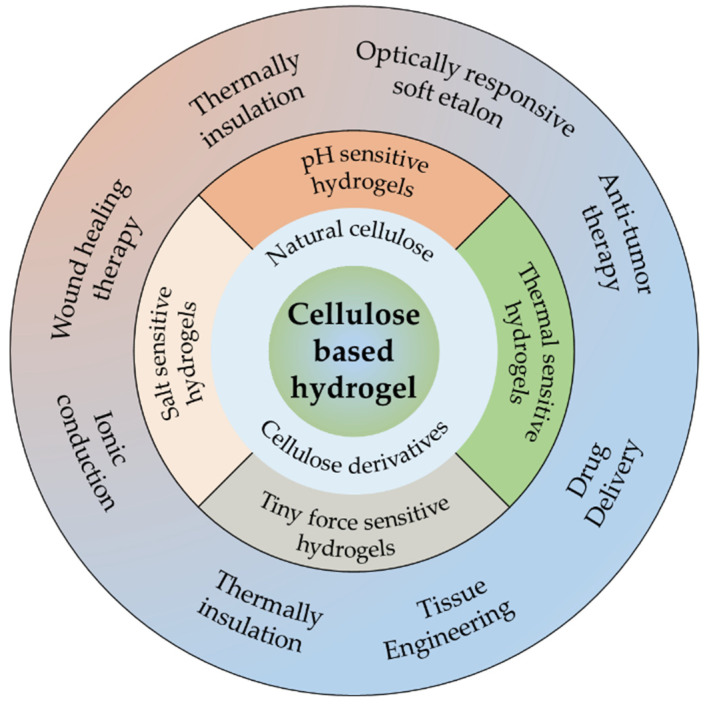
The recent advances of cellulose hydrogels in this review.

In the present review, the recent advances in cellulose hydrogels are highlighted (Figure 1). Moreover, the sources and types of cellulose, the mechanism of hydrogel formation, the research progress of hybrid cellulose hydrogels, and the different functional types of cellulose hydrogels are mainly discussed. Finally, the application and potential challenges of cellulose-based hydrogels are outlined, and future research directions are considered.

## 2. Cellulose

**Figure 2:**
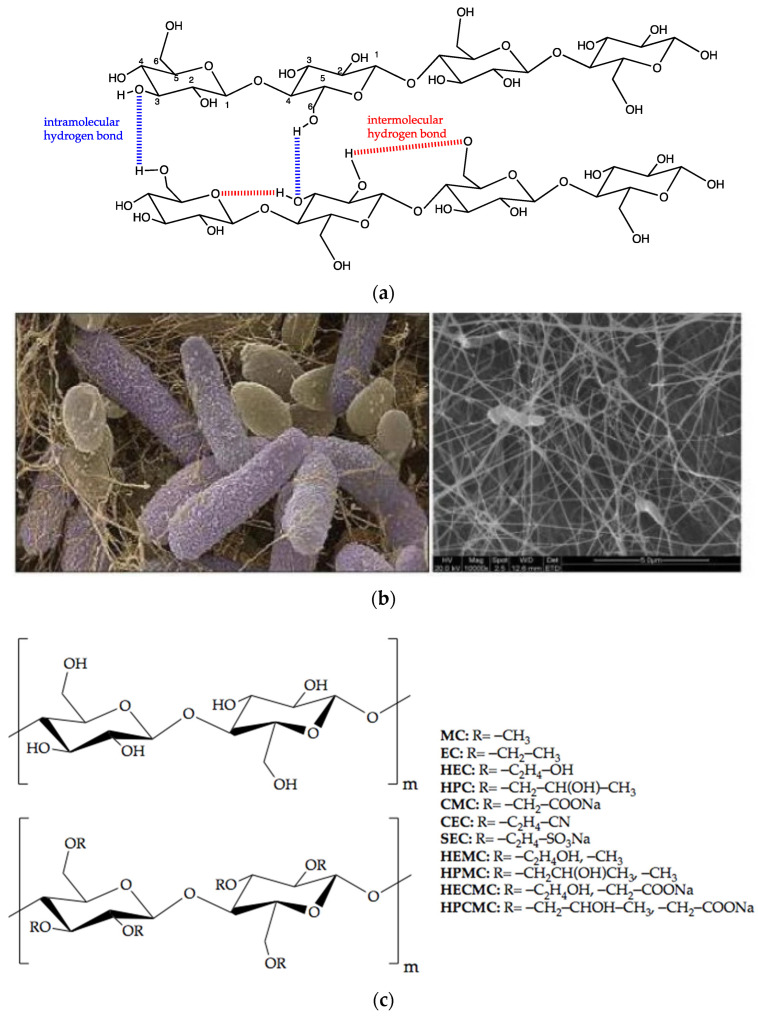
Chemical structure of cellulose and its derivatives. (**a**) Chemical structure of cellulose. (**b**) Scanning electron micrograph images of *Acetobacter xylinum* and formation of bacterial cellulose. Adapted with permission from Ref. [43] Copyright 2019, Springer Nature. (**c**) The chemical structure of cellulose and some of its derivatives. Adapted with permission from Ref. [54] Copyright 2016, WILEY-VCH Verlag GmbH & Co. KGaA, Weinheim.

As we know, cellulose was first isolated by French scientist Anselme Payen in 1838 [32]. Subsequently, the polymer form of cellulose was identified by German chemist Staudinger in 1932. Up to now, cellulose has already been extracted from readily available natural resources (such as bacteria, bamboo, jute, algae, biofilm, wood, cotton, hemp, and other plant-based materials), and is the most abundant natural macromolecular compound in the world. Five thousand to fifteen thousand glucose molecules with the molecular formula (C_6_H_10_O_5_)_n_ are covalently bonded together through C1 of the glucose ring and C4 of the adjacent ring (Figure 2a) [10,42], covalently bonded together by the acetal oxygen to form D-glucose with β-1,4 glycosidic bond [43,44]. The structure and size of natural cellulose are different in various sources, and the structural form of cellulose nanomaterials depends on processing technology. In all these multiscale materials, the structure of cellulose is extremely important because it directly affects its mechanical properties [45]. In addition, the polyhydroxy groups in cellulose can produce various forms and different functional properties after specific physical or chemical modification [46]. Therefore, the development of functional cellulose derivatives has great potential, as a means of improving the flexibility and feasibility of cellulose.

### 2.1. Classification of Cellulose

#### 2.1.1. Natural Cellulose

Cellulose is divided into native cellulose and synthetic cellulose according to its source (Table 1) [47]. Natural cellulose is composed of plant and bacterial cellulose (BC) (Figure 2b) [43,48]. Plant cellulose widely exists in cotton, wood, and other plants, such as phloem fiber, seed fiber, and wood fiber, which is the most abundant organic substance in nature [45]. Bacterial fiber refers to the cellulose synthesized by a specific species of microorganisms under different conditions, and it is the finest nano-scale fiber in nature. Multiple microorganisms can synthesize cellulose, such as *Pseudomonas* and *Acetobacter* [49]. Importantly, diverse bacteria produce cellulose with various morphology, structures, characteristics, and functionalities. For example, cellulose was secreted by some fungi and green algae (e.g., *Valonia ventricular*, *Glaucocystis*), and contained the outer cell membrane of some marine ascidians.

A wide range of studies has been conducted on the potential advantages of bacterial and plant cellulose as biomaterials [50]. Compared with plant cellulose, BC has high crystallinity and purity, because it does not produce lignin, hemicellulose, and other accompaniments [51]. It was demonstrated that the superior performance of BC satisfied the essential requirements of indispensable and versatile biomedical materials for all the practical and innovative applications [52], such as tissue engineering and wound repair [49]. The wide biomedical applications of BC are supported by the ease of production, lack of contaminants, and the capability of modulating the material’s features during syntheses—such as crystallinity index, aspect ratio, and morphology to perfectly fit the final application requirements [52]. Significantly, natural cellulose-based hydrogels were prepared from pure cellulose solutions by physical cross-linking due to the presence of numerous hydroxyl groups, which can connect the polymer network via hydrogen bonding [28]. Moreover, plant cellulose and bacterial cellulose differ in terms of macromolecular properties. Plant cellulose has a medium water-holding capacity of 60%, and a moderate level of tensile strength and crystallinity. At the same time, BC is chemically pure, hydrophilic, and it has a high water-holding capacity (100%) [52]. However, natural cellulose has multiple shortcomings such as poor solubility, low thermoplasticity, strong hydrophilicity, weak adsorption capacity, and difficult processing, which limits its development and application in the biomedical and pharmaceutical fields [53]. Fortunately, insolubility can be overcome by obtaining cellulose derivatives through various chemical modification procedures, such as esterification, etherification, or oxidation (Figure 2c) [54].

**Table 1:** Summary of the cellulose classification, property and usage.

Classification	Name	Property	Usage	Ref.
Natural cellulose	Plant cellulose	thermal and mechanical degradation; major components of plant cell walls.	fabrics, ropes, tapes, isolating materials.	[47]
Bacterial cellulose	BC	___	dura mater replacement, diagnostic sensors, dental grafting, artificial cornea, wound dressing, drug delivery system, bone tissue engineering.	[49,55]
Cellulose derivatives	Phosphate cellulose	___	enrichment agents, ion exchangers.	[56]
	Nitrocellulose	flammability, bonding.	coatings, adhesives, cosmetics, food packagings, centrifugation tube materials.	[57]
	Cellulose acetate (CA)	biodegradable, renewable, non-corrosive, non-toxic, biocompatible.	nanocomposites for biomedical applications and equipment.	[58]
	Methylcellulose (MC)	high water retention, thermogelation, macro-phase separation, syneresis.	slow-release preparation.	[59]
	Ethylcellulose (EC)	thermoplastic, water insoluble, nonionic, thermally stable, hydrophobicity.	controlled release formulations, coating agents.	[28,60]
	Carboxymethyl cellulose (CMC)	hydrophilic, bioadhesive, non-toxic, pH sensitive, thermally stable.	hindering crystallization or degradation of the drug; enhancing the frequency of drug release.	[28,61]
	Hydroxyethyl cellulose (HEC)	suspension, adhesion, emulsification, dispersion, moisture	food stabilizers, thickeners, adhesives, pharmaceutical excipients, stabilizers, film coating agents.	[44]
	Hydroxypropyl cellulose (HPC)	biodegradable and biocompatible, self-repairing abilities, shape memory, unique hydrophilic/hydrophobic change.	thermo-responsive hydrogels.	[62]
	Hydroxypropyl Methylcellulose (HPMC)	viscous soluble fiber, high viscosity, gelling.	thickener, emulsifier, stabilizer, gelling agents, antioxidants, hypoglycemics.	[63,64]
	Hydroxyethyl Methylcellulose (HEMC)	water solubility, thermally stable, gel properties.	hypoglycemics, antioxidant, coatings, medical dressings.	[64,65]

#### 2.1.2. Cellulose Derivatives

Cellulose derivatives are mainly obtained by two methods including physical and chemical modification (Table 2).

1. Physical modification

On the one hand, the physical modification is mainly used to obtain new properties and functions by changing the structure and surface properties of cellulose. In short, the physical modification mainly includes mechanical grinding, swelling, recombining, and surface adsorption without changing the chemical composition of cellulose, such as regenerated cellulose (the material obtained after cellulose is dissolved and precipitated) [66], membrane cellulose, microcrystalline cellulose, spherical cellulose [67], and nanocellulose (NC) [68].

Specifically, NC is attracting more attention because it is easy to form a self-assembly structure with stable mechanical energy. They are mainly classified into two categories: cellulose nanocrystals (CNC) and cellulose nanofibers (CNF) [69]. NC has high specific surface area, easy modification, biodegradability, non-toxic biocompatibility, wound-healing characteristics, and antibacterial effects, making it widely used in biomedical fields. For example, Gonzalez reported polyvinyl alcohol (PVA)/CNC composite hydrogels prepared by the freeze-thaw technique. Compared with pure PVA hydrogel, the obtained composite hydrogel maintains transparency, improves thermal stability, and improves mechanical properties [70].

On the other hand, the physical modification is also used to obtain new properties and functions by using inorganic salt solutions such as CaCl_2_, ZnCl_2_ or MgCl_2_. Attributable to the strong binding between metal ions and organic groups, hydrogels are endowed with unique properties such as temperature sensitivity, pH sensitivity and optical properties [71].

2. Chemical modification

The chemical modification includes two types of reactions: degradation of cellulose and derivatization of hydroxyl groups [72]. Among them, degradation reactions include acid/base degradation [73], oxidative degradation [74], biodegradation [75], and mechanical processing degradation. Derivatization, one of the most common methods for modification of cellulose, was used to synthesize derivatives of cellulose. For example, cellulose esters, such as cellulose nitrate, cellulose xanthate, cellulose acetate [58], cellulose acetate phthalate (CAP), and hydroxypropylmethyl cellulose phthalate (HPMCP), are synthesized by esterification of hydroxyl groups with various organic acids in the presence of a strong acid as a catalyst [76]. Cellulose derivatives obtained by esterification have good properties of film-forming, which are suitable for standard coatings. Moreover, it was essential for drug-delivery applications that cellulose esters are not only non-toxic and stable, but are not absorbed from the gastrointestinal tract [28].

Significantly, hydroxyl derivatization (including nucleophilic substitution, graft copolymerization, and cross-linking reactions) is beneficial to improve the water solubility of cellulose. A classic example is cellulose ether, a class of polymer compound with an ether structure—such as methyl cellulose (MC), hydroxypropyl methyl cellulose (HPMC), hydroxyethyl cellulose (HEC), and carboxymethyl cellulose (CMC), in which the hydrogen of the cellulose hydroxyl group is replaced by the hydrocarbon group [46]. Compared with cellulose, cellulose ether has excellent advantages of water-solubility and thermoplasticity, which is significant for the preparation of hydrogels. Therefore, it is expected that the mechanical strength, hydrophobicity, heat resistance, and antibacterial activity of cellulose can be improved by modification, and the application range of cellulose can be expanded [44].

In addition, graft copolymerization is also a hot topic in the research of cellulose modification. Ring-opening grafting of cyclic monomers (e.g., epoxides, lactones, cycloimines, cyclic-thioethers) is one of the commonly used methods for graft modification of cellulose. Different flexible groups can be introduced by grafting modification to plasticize the internal cellulose. This method retaining the inherent properties of cellulose endows it with thermoplasticity [54].

### 2.2. Property of Cellulose Materials

Natural cellulose is neither soluble in water nor in common organic solvents such as ether and acetone at room temperature. To dissolve cellulose, two methods are mainly used in industry: the derivatization method [77] and the direct method [78]. The derivatization method generally swells cellulose in sodium hydroxide and activates it to produce cellulose derivatives which are dissolved in alkali. A special solvent system is used to dissolve cellulose directly without modifying the property of cellulose by the direct method [79] In addition, the strong intramolecular and intermolecular hydrogen bonds of cellulose endue cellulose with many special properties, such as crystallinity, water absorption, self-assembly, and chemical activity, electrical conductivity, thermal conductivity, and optical properties [80].

#### 2.2.1. Mechanical Properties of Cellulose Materials

Some factors, such as crystal structure (Iα, Iβ, II), crystallinity percentage, anisotropy, and performance measurement methods and techniques may affect the measured mechanical properties. The mechanical properties, mainly the elastic properties of several cellulose particles, have been reported previously [42,81]. Here, we have briefly summarized them. In short, there are four basic parameter influenced properties of cellulose-based materials: elastic modulus in the axial direction (E*_A_*), the elastic modulus in the transverse direction (E*_T_*), tensile strength (σ*_f_*, tensile testing), and strain to failure (ε*_f_*, tensile testing). The elastic properties have been measured using in situ tensile tests combined with XRD and inelastic X-ray scattering (IXS) to measure strain [42].

#### 2.2.2. Conductive Properties

Hydrogels are composed of polymer networks and more than 90% water, and therefore can be used as ideal ionic conductors when mixed with electrolyte salts. However, the polymerization process is time-consuming and energy-consuming, and the poor adaptability to the extreme environment has seriously hindered its development in the emerging green power field. Therefore, it is still a challenge to prepare ionic conductive hydrogels with high mechanical properties, high electrical conductivity, and good freezing resistance through a simple method.

For example, Wang directly generated ionic conductive cellulose hydrogels (CCHs) with antifreeze properties through a simple one-step chemical cross-linking [82]. Cellulose was dissolved in an aqueous solution of benzyl trimethyl ammonium hydroxide (BzMe_3_NOH), and CCHs were directly obtained by chemical cross-linking without further treatment. The conductive hydrogel is endowed with sensitivity, high transparency, and elasticity by the solvent, and can still maintain a stable performance at low temperatures. These advantages make the cellulose-based hydrogels show promising application prospects in sensors, energy storage, and wearable devices at sub-zero temperatures.

#### 2.2.3. Thermal Properties

Typically, thermochemical degradation of cellulose occurs between 200–300 °C, depending on the heating rate, particle type, and surface modification type. Iα is composed of single-chain triclinic monomer units, and Iβ is composed of double-chain monoclinic monomer units. The former is mainly cellulose in green algae and bacteria, and the latter is the main crystalline form of cellulose in wood and cotton. This also leads to a certain anisotropy of the thermal conductivity of cellulose [24]. The thermal conductivity of cellulose determines its important position in hydrogel-based thermal insulation materials. For example, Zhang et al. integrated ionic compounds (ZnCl_2_/CaCl_2_) into cellulose hydrogel networks to enhance frost resistance [83]. Their specially designed ZnCl_2_/CaCl_2_ system has excellent freezing resistance and improved the solidification rate of cellulose by extra water or glycerol.

In addition, MC is a typical temperature-responsive water-soluble polymer [84], which can form gels in the presence of salt at 37 °C. Inspired by this criterion, MC has become an attractive candidate for the preparation of thermosensitive physical hydrogels in the field of drug delivery, tissue engineering, etc. [85,86]. For example, a thermo-responsive MC hydrogel was prepared by crosslinking with citric acid for cell sheet engineering, allowing cell detachment from their surface by lowering the temperature below transition temperature [87].

#### 2.2.4. Optical Properties

Anisotropic structures have unique characteristics in many aspects, such as electricity, mechanics and optics, and most biological soft tissues (muscle, skin and cartilage) have multi-layered ordered structures ranging from nano scale to macro scale, which play a crucial role in organisms [88]. As a kind of soft and wet material, hydrogels are widely used in biomedical materials, flexible electrodes, brakes, sensors, etc. Compared with traditional hydrogels, anisotropic hydrogels with oriented structures have greater potential in all aspects [89]. However, the preparation of highly oriented hydrogels remains challenging because the regulation of macromolecular chains is often limited by electrostatic, hydrogen bonding and other interactions.

**Figure 3:**
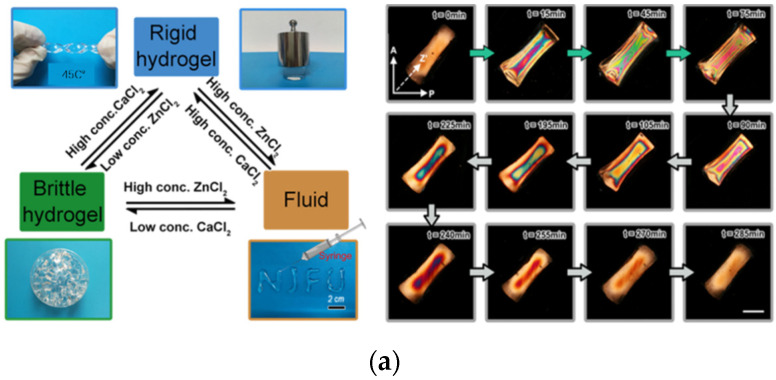

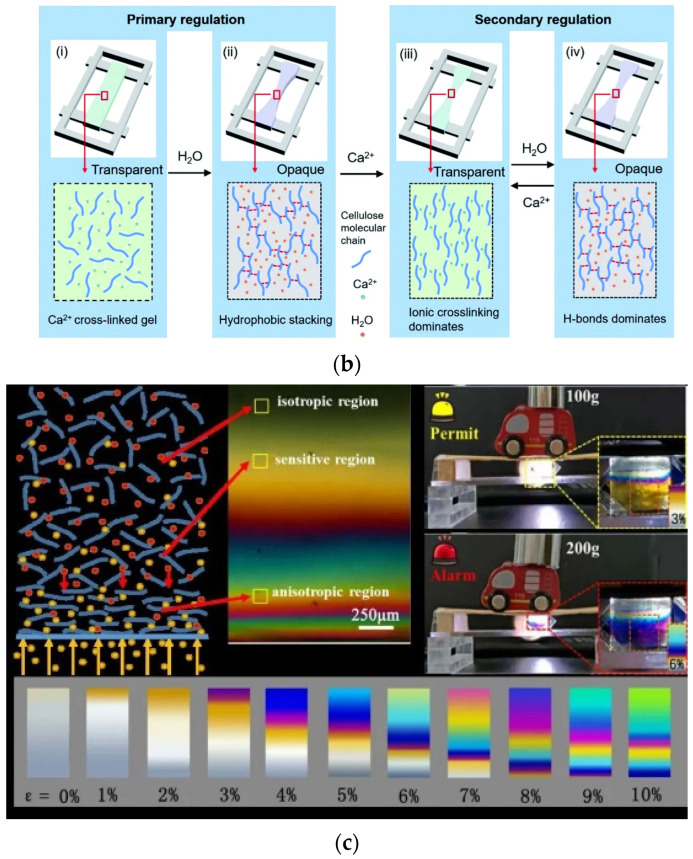
A new method for the preparation of anisotropic cellulose hydrogels induced by calcium ions. (**a**) Reversible multiphase transformation of Cellulose hydrogels based on Ca^2+^/Zn^2+^ exchange and color change of the gel interference under orthogonal polarized light during ion exchange. Adapted with permission from Ref. [71] Copyright 2020, WILEY-VCH Verlag GmbH & Co. KGaA, Weinheim. (**b**) The schematic diagram of the preparation of a highly oriented cellulose hydrogel by H_2_O/Ca^2+^ exchange shows that the flexible switch between ion coordination/hydrogen bond dominance is achieved, thus achieving continuous regulation of a high-oriented structure. (i) The initial gel cross-linked by Ca^2+^. (ii) The hydrophobic stacking of cellulose chains triggered by water along the length direction to form an aligned structure. (iii) Ca^2+^ breaks the H-bonds between the cellulose chains and is simultaneously cross-linked with cellulose molecules. (iv) Hydrophobic stacking occurred again when the gel was soaked in water. With the repeated H_2_O and Ca^2+^ exchange process, the cellulose molecular chains continued to adjust along the confined direction, resulting in a high orientation structure. Adapted with permission from Ref. [90] Copyright 2020, WILEY-VCH Verlag GmbH & Co. KGaA, Weinheim. (**c**) Mechanism diagram and microstrain test diagram of gradient anisotropic hydrogel prepared by directional diffusion of cellulose sol in CaCl_2_ solution. Adapted with permission from Ref. [91] Copyright 2021, Elsevier Ltd.

Based on the regenerated cellulose hydrogels prepared by ZnCl_2_/CaCl_2_ dissolution system in the early stage [83], He Group conducted a series of studies on the preparation of anisotropic cellulose hydrogels induced by Ca^2+^ ions. For example, a simple method for the preparation of multiphase convertible cellulose hydrogels under Ca^2+^/Zn^2+^ ion exchange was reported (Figure 3a) [71]. The introduction of Ca^2+^ can not only improve the compressive strength of cellulose hydrogel, but also improve the orientation of the hydrogel through compression setting. The ionic hydrogel has good pressure response performance and can effectively monitor the slight bending of fingers and pressure changes. In addition, a new method for producing cellulose hydrogels with high orientation (refraction index: ~6.4 × 10^−3^) and high-water content (~72%) was proposed (Figure 3b) [90]. This strategy uses Ca^2+^ coordination cycles to break the hydrogen bonds between cellulose chains, and flexibly switches between ion coordination/hydrogen bond dominance, achieving continuous regulation of high-orientation structures. This principle may provide a new way to construct highly oriented structures and prepare a variety of stimulus-response anisotropic materials. Subsequently, they also prepared gradient anisotropic cellulose hydrogels by the CaCl_2_ solution diffusion method [91]. The orientation of the cellulose chain in the hydrogel shows a characteristic of decreasing along the direction of ionic diffusion. They proved for the first time the concept of sensitive regions in the ordering-disorder transition region of gradient hydrogels. On this basis, a readable strain response colorimetric card that can be used to detect tiny strains was designed (Figure 3c). This strategy has great potential in the fabrication of optical response devices, complex 3-D structures, and bionic structures.

In addition, under the action of external forces, the elastic chemical crosslinking network can ensure the large deformation of hydrogels, while the fracture and rearrangement of the physical crosslinking network can effectively dissipate energy, endowing cellulose hydrogels with good strength, toughness and force-induced optical anisotropy. For example, the Zhang Group prepared a novel cellulose hydrogel with a small amount of epichlorohydrin (EPI) in LiOH/urea solution and subsequent treating with dilute acid by using cellulose cotton short pulp to show sensitive force-induced optical anisotropy properties [92]. It was confirmed that the unique structure endows the hydrogel with excellent mechanical properties and the force-induced optical anisotropy is derived from the force-induced structural orientation. This kind of cellulose hydrogel can be designed as an intelligent soft matter force sensor to sense external forces because of its sensitive force-induced optical anisotropy behavior.

## 3. Preparation Methods of Cellulose Hydrogel

**Figure 4:**
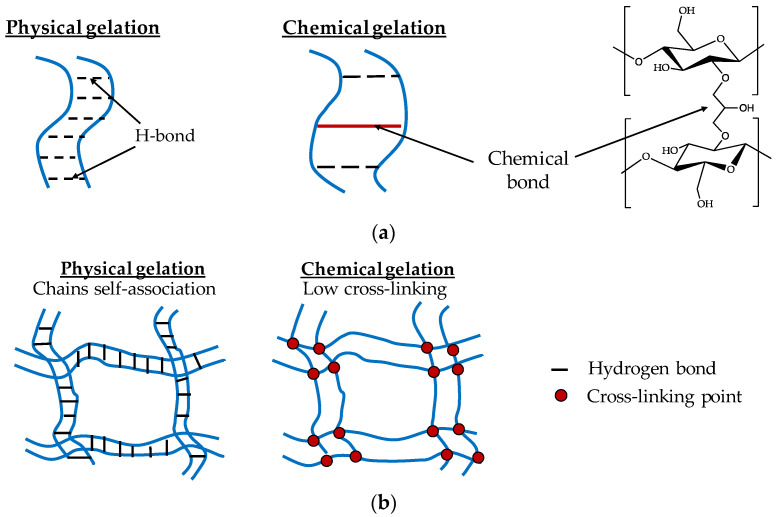

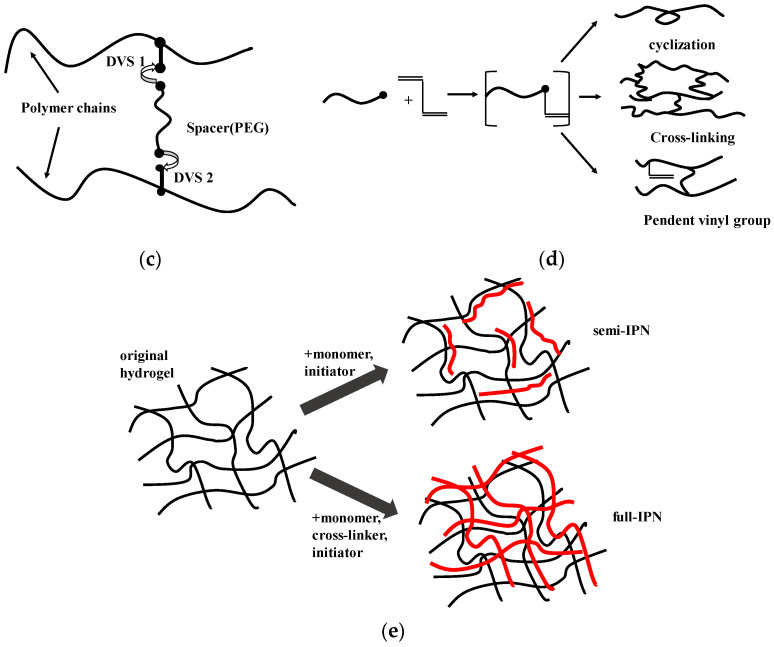
Formation mechanism of hydrogel. (**a**) A sketch of network formation in cellulose solutions: physical gelation via self-association of chains and chemical cross-linking; (**b**) a schematic presentation of the structures of physical and chemical cellulose gels; adapted from Ref. [28]. (**c**) Scheme of the crosslinking between celluloses in the presence of spacers. DVS: divinyl sulfone, cross-linked molecules. (**d**) Synthesis route of hydrogel by radical polymerization. Adapted with permission from Ref. [99] copyright 2007, Society of Chemical Industry. (**e**) Formation and structure of semi- and full interpenetrating hydrogels. Adapted with permission from Ref. [103] Copyright 2008, Elsevier Ltd.

A cellulose-based hydrogel is prepared by physical or chemical cross-linking (Figure 4a,b) [28]. The hydroxyl groups of cellulose and various groups after modification are beneficial to the cross-linking of cellulose to obtain cellulose-based hydrogels (Table 3). The preparation of hydrogels is closely related to the formation of its interconnected network structure. Interactions of physical properties include mechanical chain entanglements, van der Waals interactions, hydrogen bondings, hydrophobic force aggregations or electronic associations. Mastering the preparation principle and method of hydrogel will help us to better design biomedical materials that could meet different needs. In this section, we will briefly introduce the preparation methods of hydrogel, including physical, chemical cross-linking, and interpenetrating networks.

### 3.1. Physical Cross-Linking

Physical hydrogels, also called pseudo hydrogels or thermally reversible hydrogels, are formed through van der Waals forces, hydrogen bonds, ionic bonds, and hydrophobic interactions. The interaction between physical cross-linked cellulose hydrogel molecules is reversible, and the network structure is destroyed with the change in physical conditions [93]. Physical gelation is the self-association of the cellulose chain. Cellulose preferentially binds to cellulose rather than to cellulose solvent, which is often accompanied by a microphase separation. Physical cross-linking methods have the following four mechanisms: (1) interaction between ions: the physical cross-linking network based on PVA and chitosan (CS) was mainly constructed by immersing the Na_3_Cit solution as the dynamic bond, and the N-glucosamine unit of the CS chain in the tridentate coordination of the Cit^3−^ anion [94]; (2) crystal cross-linking: Koichi proposed a non-damage enhancement strategy for hydrogels using strain-induced crystallization [34]. For the highly oriented slip-ring hydrogels with polyethylene glycol chains exposed to each other under large deformation, crystallization formed and melted with elongation and contraction, resulting in almost 100% rapid recovery of tensile energy and excellent toughness of 6.6 to 22 megajoules each square meter; (3) hydrogen bond cross-linking: there is a new strategy to dynamically adjust the hydrogen bond cross-linking between PVA and tannic acid (TA) by ethanol, which can be simply coated on the surface of porous substrates by different methods [95]; and (4) hydrophobic association: Sun made the short alkyl side chain modified hydrogel library into a model phase separation hydrogel [96]. With the increase of side chain length, stronger hydrophobic interaction was generated. The longer side chain in the hydrogel promoted a thicker and denser network. Therefore, the layering was faster compared with the short side chain.

### 3.2. Chemical Cross-Linking

Different from physical hydrogels, chemical hydrogels are irreversible with three-dimensional networks connected by covalent bonds. The starting material can be a monomer, polymer or a mixture of monomer and polymer (Figure 4c) [97]. What’s more, the preparation process is not spontaneous; on one hand, the reaction is triggered by radiation. On the other hand, the polymer reacts with small molecule cross-linking agents. Chemical cross-linking hydrogels are prepared by functional coupling agents or by cross-linking more than two polymer chains under ultraviolet light. Hydrogels prepared by this method will be more stable and have better swelling performance [98]. Various cross-linking agents and catalysts are used for the chemical cross-linking of cellulose derivatives. The most commonly used cross-linking agents are dialdehydes, acetals, polycarboxylic acids, epichlorohydrin and polyepichlorohydrin. The chemical cross-linking method disrupts the self-association and packing of cellulose leading to swollen transparent coagulated cellulose hydrogels with more uniform morphology and porous structure, lower crystallinity, higher swelling and higher water vapor adsorption affinity [43]. Chemical cross-linking includes radiation polymerization and free radical polymerization (Figure 4d) [99]. For instance, a polyacrylamide/acidified single-walled carbon nanotube composite hydrogel was used to assemble quasi-solid TEC by Chen [100]. The hydrogel was chlorinated with Sn (IV)/Sn (II) chloride (Sn^4+^/Sn^2+^) as redox pairs, and a simple in-situ free radical polymerization route was used to fabricate a hydrogel with high thermos electro chemical Seebeck Coefficient and excellent thermos electro chemical stability against large mechanical tensile and deformation [101]. In addition, a polyacrylamide-based hydrogel sensor was also designed by Wen, where hydroxyl radicals are caused by the radiolysis of X-ray water molecules [100].

In addition to using the above two methods alone, physical and chemical cross-linking can also be used together. For example, Zhao proposed a strategy using sequential chemical cross-linking and physical cross-linking to form mechanically strong and tough double-crosslinked (DC) cellulose hydrogels from cellulose/alkali hydroxide/urea aqueous solutions [102]. The resulting DC cellulose hydrogels were transparent, foldable and were elastic and featured quick recovery properties. Moreover, the nanostructured network of DC cellulose hydrogels was achieved by covalent cross-linking, hydrogen bonding and chain entanglement between cellulose chains and cellulose II microcrystalline hydrates in DC cellulose hydrogels. The hydrogel effectively improved the mechanical properties of DC cellulose hydrogel, which exceeded the single chemical cross-linking and physical cross-linking cellulose hydrogel. The hydrogel developed in this work represents the first example of a cellulose hydrogel with high strength and toughness, whose excellent mechanical properties stem from the cross-linked structure resulting from a sequential chemical and physical cross-linking strategy. DC cellulose hydrogels are a new class of polysaccharide hydrogels with potential applications in artificial blood vessels and skin, tissue engineering materials, and catalyst carriers.

### 3.3. Interpenetrating Network

An interpenetrating network polymer (IPN) is an aggregation structure formed by the penetration of two polymers in the form of a network (Figure 4e) [103,104]. One of the polymers is chemically cross-linked and the other penetrates its network of it, so that there is no covalent bond between the two polymers [105]. Cellulose-based hydrogel prepared by the IPN method was a two-component network structure, which has the characteristics of a large swelling ratio and good mechanical properties. It has good application prospects in biomedical tissue engineering, adsorption, and separation [106].

**Table 3:** Classification of hydrogel crosslinking methods.

Crosslinking Types	Crosslinking Mechanism	Crosslinking Methods	Reference
Physical crosslinking	Ions interaction	Based on polyvinyl alcohol, a physical cross-linked network was constructed by coordinating the N-glucosamine unit of chitosan chain with the tridentate ligand of Cit^3^^−^ anion.	[94]
	Crystalline crosslinking	Crystallization formed in polyethylene glycol chain slip ring gel and melted with elongation and contraction.	[34]
	Hydrogen bonding crosslinking	Between PVA and Tannic Acid (TA).	[95]
	Hydrophobic association interaction	Increased side chain length leads to stronger hydrophobic interactions and promotes thicker and denser networks.	[96]
Chemical crosslinking	Free radical polymerization	Composite hydrogel formed by in situ radical polymerization of poly-acrylamide/acidified single-walled carbon nanotubes.	[101]
	Radiation polymerization	Polymerization of monomers is initiated by high-energy radiation.	[100]
	Interpenetrating polymer network	PVA network with high crosslink density (a skeleton restricting the saturated water content of gel) is penetrated by polystyrene sulfonate (PSS) network.	[107]

The semi-interpenetrating network (Semi-IPN) hydrogel is synthesized by immersing the monomer and initiator into the hydrogel solution, and the full-IPN hydrogel is formed by adding cross-linking agent [103]. Compared with hydrogels prepared by other methods, hydrogels prepared by the interpenetrating network method have good mechanical strength, better flexibility, controllable physical properties, and more effective drug loading. Since the micropores are adjustable, the release behavior of drug conforms to controlled release kinetics [108]. For example, a simple strategy to control the hydration of polymer networks in hydrogels was reported by Zhao, in which highly skeletonized polymer networks can be used to functionalize densely cross-linked polymers as skeletons [109]. The hydration of polymer chains will produce a large number of weakly bound water molecules, thereby promoting water evaporation. A PVA network with a high cross-link density (a skeleton restricting the saturated water content of hydrogel) is penetrated by a polystyrene sulfonate (PSS) network which can be actively hydrated by water molecules through electrostatic and hydrogen bonds. Hydrogels with this interpenetrating polymer network can activate more than 50% of the water into an intermediate state.

## 4. Cellulose-Based Hydrogels

Cellulose-based hydrogels as biomaterials should be biocompatible. In the meantime, it should perform special biochemical, mechanical, and physical properties in order to simulate fundamental aspects in vivo. Biocompatibility signals the likelihood of a material to coexist and interact without harm in the presence of a specific tissue or biological function. Assessing the biocompatibility of a biomaterial requires examining the harm or side effects it may cause to the host. The feature of the cellulose-based hydrogels, such as biocompatibility, biodegradability, adjustable mechanical properties, sensitivity to various stimuli, the ability to encapsulate different therapeutic agents and to control drug release make it an important candidate for biomedical applications.

### 4.1. CNF-Based Hydrogels

CNF-based hydrogels, which have three-dimensional NF networks and unique physical properties, have great applications in elastic hydrogels, ionic conduction, and water purification for emerging materials [41]. CNF is composed of a cellulose chain, which is long and flexible and arranged in the structure controlled by hydrogen bonds. CNF is characterized by a recognized cytocompatibility and a high tolerogenic potential. For example, Hong demonstrated the gelation of carboxylated cellulose nanofibers and the formation of interconnected porous networks by adding divalent or trivalent cations (Ca^2+^, Zn^2+^, Cu^2+^, Al^3+^, and Fe^3+^) to aqueous nanofiber dispersions. Dynamic viscoelastic measurement showed that the hydrogel modulus can be adjusted by appropriate selection of cations. Gelation was induced by screening for repulsive charges on the nanofibers, and gel properties were controlled by ionic crosslinking. We can envision multiple potential applications of CNF-Mn+ hydrogels in biomedicine and other fields, such as drug carriers and the encapsulation of functional molecules.

### 4.2. Cellulose-Based Hybrid Smart Hydrogels

As we know, the poor mechanical properties of traditional biomolecular hydrogels severely limit their application prospects. Traditional hydrogels have been increasingly unable to meet the growing needs of biomedicine. Smart hydrogels (stimuli-responsive hydrogels) have attracted extensive attention in academic and industrial fields due to their high elasticity and adaptability, which can respond to environmental stimuli rapidly and significantly [110]. Therefore, how to prepare smart biocompatible hydrogels with high strength, high toughness and high-water content from biological macromolecules is a hot research topic in hydrogel fields [111,112].

According to the response to external stimuli, intelligent polymer hydrogels can be allocated to pH sensitive hydrogels, thermal sensitive hydrogels, salt sensitive hydrogels, tiny force sensitive hydrogels, and so on [113].

#### 4.2.1. pH Sensitive Hydrogels

pH sensitive hydrogels contain a large number of acid and base groups that are easily hydrolyzed or protonated, such as carboxyl and amino groups. When the external pH value changes, the degree of protonation or deprotonation of these groups changes accordingly, resulting in changes in the electrostatic attraction or repulsion between functional groups, and the swelling degree of hydrogels [114]. At the same time, with the change of pH value, the difference in ion concentration inside and outside the hydrogel also changed, resulting in the change of osmotic pressure inside and outside the hydrogel, and the change in swelling degree of the hydrogel [115]. In addition, the dissociation of these groups will also destroy the corresponding hydrogen bonds in the hydrogel, reduce the cross-linking points of the hydrogel network, cause a change in the hydrogel network structure, and cause hydrogel swelling [116]. For example, polyacrylic acid, polyacrylamide, and chitosan are typical pH sensitive polymer hydrogels. Yin et al. prepared an intelligent pH sensitive hydrogel based on the oxidation of hydroxyethyl cellulose from pineapple peel and the residual carboxymethyl chitosan from Hericium Erinaceus. It was done mainly through the oxidation and alkalization of cellulose hydrogels. Cellulose was modified to oxidized dialdehyde cellulose, and chitin was modified to CMC, which was combined by a Schiff base reaction to form a new hydrogel with excellent characteristics [117].

#### 4.2.2. Thermal Sensitive Hydrogels

In 1978, Tanaka et al. first found the thermal sensitivity of hydrogels when they studied polyacrylamide [118]. The thermal sensitivity of hydrogels refers to the volume mutation that occurs with the change in ambient temperature. The hydrogel has a certain proportion of hydrophobic and hydrophilic groups. The temperature change can affect the hydrophobic effect of these groups and the hydrogen bond between macromolecular chains so that the gel structure changes and the volume changes.

Inspired by this criterion, cellulose derivatives, such as MC, HPC, HPMC, and EHEC, have become an attractive candidate for the preparation of thermosensitive physical hydrogels. For example, a thermoreversible hydrogel was prepared by combining high molecular weight hyaluronic acid with rapidly oxidized nanocellulose, MC and polyethylene glycol to prevent adhesion. Among them, MC ensured the thermal sensitivity of this hydrogel [119].

As thermal responsive hydrogels, HPC-based hydrogels have also been studied in depth. HPC hydrogels respond to temperature changes by volume phase transition. Below the phase transition temperature (T_t_ = 41 °C), the hydrogel is hydrophilic, swelling in water. The hydrogel becomes hydrophobic, and disintegrates into a small volume when the temperature is above the T_t_ [120]. Also, HPMC is a polysaccharide derivative with water solubility, pH stability, biodegradability and biocompatibility [121]. Due to the dehydration of the hydrophobic substitution zone of the polymer chain, HPMC can undergo thermal reversible sol-gel phase transition during heating. Although the gel temperature of HPMC was 60 °C, much higher than 37 °C, Wang destroyed the polymer water sheath by adding a high concentration of glycerol, promoting the formation of a hydrophobic region and reducing T_t_. Furthermore, EHEC has a low critical dissolution temperature, and phase separation occurs above this temperature. With the increase in temperature, EHEC becomes more hydrophobic and induces the formation of large aggregates separated from the water phase. Specifically, ionic surfactant was added to EHEC, which significantly changed this situation and formed low-toxic thermoresponsive hydrogels [122].

In addition, the thermal response of CNF-based hydrogels can also be triggered intelligently. For example, Wei et al. prepared an ionic skin–water solvent network based on high-performance organic hydrogels with superior mechanical response and thermal sensing ability through one-step UV-initiated polymerization, which can be assembled into capacitive sensors for motion monitoring in real life and thermal resistance for dynamic temperature detection. In short, olyacrylamide (PAAm), CNFs, tannic acid (TA), electrolytes (NaCl), and glycerol/water binary solvent are incorporated by UV-initiated free radical polymerization [123]. In addition, based on the temperature-dependent self-association of betaine methacrylate (SBMA), polymer chains and the incorporation of temperature sensitive cellulose/polyaniline nanofibers (CPA NFs) in a glycerol-water binary solvent system, Hao et al. successfully prepared an intelligent temperature-sensing amphoteric hydrogel with superior low-temperature tolerance and conformal adhesion [124].

#### 4.2.3. Salt Sensitive Hydrogels

The swelling rate/water absorption rate of salt sensitive hydrogels will vary with the change of salt concentration outside because of its structure. The positive and negative charged groups of these hydrogels are bonded together by covalent bonds, and the addition of small molecular salts can shield and destroy the association of them in the macromolecular chain, resulting in the stretching of the molecular chain. The outstanding advantage of such hydrogels is that the swelling behavior of hydrogels in salt solution presents the anti-polyelectrolyte behavior. In another word, under certain conditions, the swelling ratio of hydrogels in salt does not decrease but increases with the increase of the applied salt concentration. For example, Hai et al. developed a novel fluorescent switch-reporting ClO^−^/SCN^−^ reversible responsive cellulose hydrogel. When ClO^−^ was added, the hydrogel network of NC hydrogel was destroyed, and the fluorescence was quenched. Hydrogel changes in a completely reversible process by regulating ClO^−^/SCN^−^ [102].

#### 4.2.4. Tiny Force Sensitive Hydrogels

Strain sensors can sense external stimulus signals and convert them into recordable electrical signals, which are widely used in human daily motion monitoring, skin perception, and other fields. Ouyang et al. prepared gradient anisotropic carboxymethyl cellulose hydrogel (CMC-Al^3+^) CMC-Al^3+^ by directional diffusion of aluminum chloride solution. The CMC-Al^3+^ was packaged with PVC flame retardant tape, and a strain sensor for detecting micromotion of the human body was made. It can accurately and stably monitor micromotion [125]. Ye et al. found that cellulose was reacted with a small amount of epichlorohydrin (EPI) in LiOH/urea solution, and then treated with dilute acid to prepare tough cellulose hydrogel with deformation-induced anisotropy. This cellulose hydrogel has sensitive mechanical response characteristics and can be used as dynamic light switches and soft sensors to accurately detect small external forces [92].

## 5. Application

During the past decades, cellulose-based hybrid smart hydrogels/aerogels have been developed in an increasing number of applications which significantly contribute to our public healthcare systems [126]. Cellulose is known to be a naturally abundant, mechanically excellent, sustainable technological, and inexpensive material [127]. Among them, naturally derived nanocelluloses possesses unique physicochemical properties and great potential as renewable smart nanomaterials, opening up a large number of new functional materials for multi-sensing applications. Cellulose not only plays a huge role by itself but synergizes with other materials to achieve the manufacture of more advanced and multifunctional hybrid materials. In recent years, many advances have been made in the design of functional materials with excellent mechanical properties using cellulose [45,128]. In this section, we will focus on the advances of cellulose-based hydrogels in biomedical applications.

### 5.1. Ionic Conduction/Battery

With the rapid development of the Internet of Things and the increasing demand for human-machine interfaces, flexible ionic conductors have attracted extensive attention due to their characteristics of high elasticity, transparency, adjustable mechanical properties, and consistent electrical conductivity. Cellulose-based conductive hydrogels for tissue engineering are constantly progressing [10]. Recently, conductive hydrogels showed great prospects for extensive and important applications in the field of sustainable energy such as sensors, batteries, and flexible electronic devices [129] because of their unique characteristics of sufficient flexibility, durability, and versatility. Traditional hydrogels lose their original properties due to freezing at low temperatures, which limits their application. For example, a freeze-resistant ionic conductive cellulose hydrogel, which can be used as a tensile, compression and temperature sensor was prepared [82]. Unlike traditional hydrogels, its frost resistance allows it to work well in sub-zero temperatures. The sensor based on antifreeze conductive hydrogel has stable mechanical property and thermal sensitivity, and fast, reliable, stable and reversible response performance. It is suitable for low temperatures, and is used for soft artificial intelligence devices in complex temperature environments. In recent years, the use of renewable resources and green preparation technologies to prepare smart hybrid materials has attracted the extensive attention of researchers. For example, the most advanced nanocellulose-graphene composites [130] can be used for multi-functional sensing platforms such as mechanical, environmental and human biological signal detection, simulation and field monitoring. Moreover, a number of researchers have also been involved in investigating the exploitation of CFs as load-bearing components for composites. The use of these materials in composites has increased due to their relative cheapness compared to conventional materials such as glass and aramid fibres, their recyclability, and the fact that they compete well in terms of strength per unit weight of material [47].

**Figure 5:**
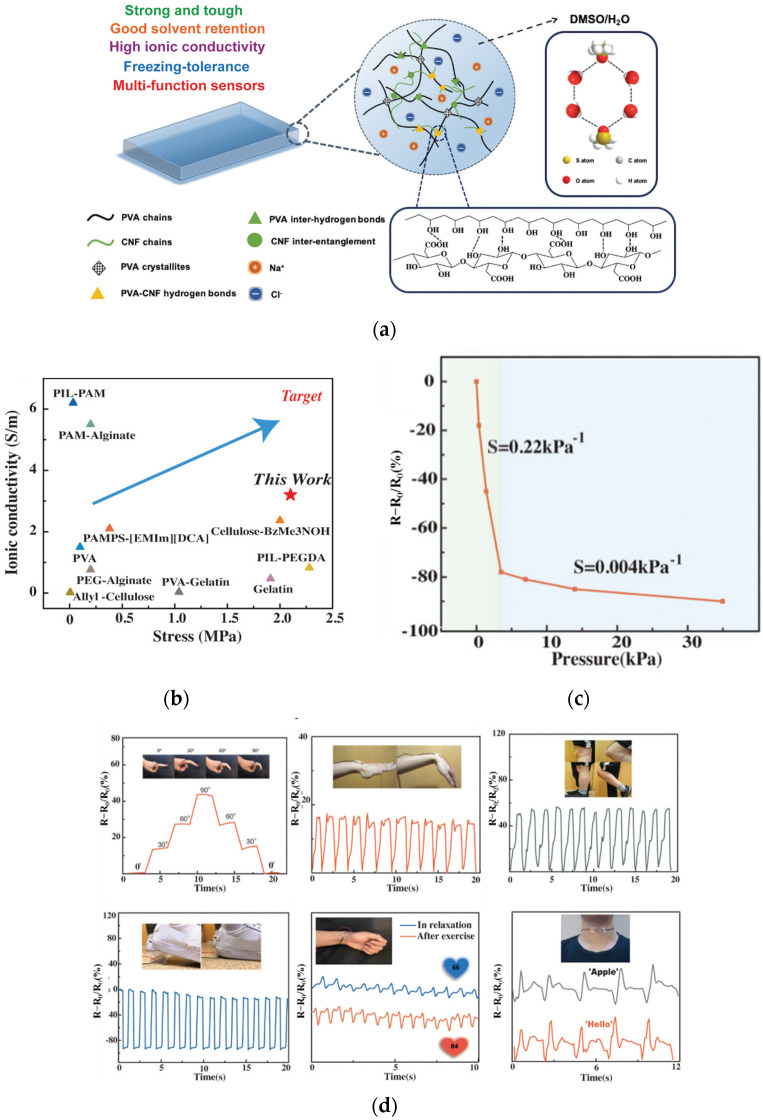
Cellulose-based ionic conductive hydrogel for multi-functional sensors. (**a**) Schematic illustration of PVA-CNF organohydrogel. (**b**) Ashby plot of ionic conductivity and tensile stress with other reported ionic conductive (organo) hydrogels. (**c**) Relative resistance changes and pressure sensitivity of PVA-1%CNF organohydrogels-based sensor at varying pressure. (**d**) The relative resistance changes of sensors versus time for real-time monitoring of various human motions. Adapted with permission from Ref. [131], WILEY-VCH Verlag GmbH & Co. KGaA, Weinheim.

Recently, a new transparent ionic conductive hydrogel with excellent mechanical properties, high conductivity, high transparency, and freezing resistance based on PVA and CNFs was prepared by the sol-gel transformation method (Figure 5a) [131]. The multi-layer porous structure of CNFs/PVA plays an important role in enhancing the ionic conductivity of organic hydrogels (Figure 5b). Pressure sensors based on conductive hydrogels with high-pressure sensitivity (Figure 5c) are used to detect complex human movements in real time (Figure 5d). In short, this material design demonstrates the synergistic effect of CNFs in improving mechanical properties and ionic conductivity, solving the long-standing dilemma between the strength, toughness, and ionic conductivity of ionic conducting hydrogels, which are widely used in wearable electronic devices.

Zinc-air batteries are regarded as the ideal power source for the next generation. Limited by the zinc-air battery electrolyte, it has rarely been reported in the improvement of flexibility and wearability. Although there have been many reports of stretchable supercapacitors and stretchable batteries, preparing ultra-long stretchable zinc-air batteries remains a major challenge. Recently, a sodium polyacrylate/cellulose double network hydrogel, which has ~1200% tensile properties in strong alkali as a result of the introduction of cellulose into the sodium polyacrylate, was prepared [132]. Based on sodium polyacrylate/cellulose network hydrogel, the planar electrode was prepared by using a wavy zinc electrode, of which the air electrode has a maximum tensile strength of 800% and the fiber electrode can be stretched up to 500%. The hydrogels with double network structure prepared by them have good alkali resistance, which can also be used in other alkaline electrolyte storage devices.

The transient device is a new electronic device whose main characteristic is that after completing the task, it can completely or partially dissolve or decompose the constituent material through a chemical or physical process, which is considered a new research direction for an implantable device. However, the research on transient devices is still in its infancy, and many challenges need to be overcome, especially since the development of transient energy devices is relatively slow. Recently, a transient zinc ion battery (TZIB) with excellent biocompatibility and complete degradation based on a carefully designed cellulose aerogel-gelatin (CAG) solid electrolyte was reported [23]. The new fully degradable CAG solid electrolyte enables TZIB to achieve controlled degradation and stable electrochemical performance while maintaining excellent mechanical properties. More importantly, TZIB has excellent electrochemical performance while meeting the requirements of controlled degradation. These results demonstrate the potential of TZIB for future clinical applications and provide a new platform for transient electronics. This is the first time that the perfect combination of high flexibility, high mechanical performance, and high biocompatibility has been achieved while maintaining high battery performance. Thus, this work offers new opportunities for future self-powered transient electronic devices or traditional self-powered implantable medical devices, such as implantable cardioverter defibrillators, implantable diagnostic sensors and the rapidly evolving implantable monitoring of diabetes. In addition, a soft stretchable conductive hydrogel composite consisting of alginate, carboxymethyl cellulose, polyacrylamide, and silver flakes was reported [133].

### 5.2. Thermal Insulation

In the next five years, cellulosic polyporous materials with thermal insulation, light weight, and excellent mechanical properties are expected to replace commercial thermal insulation materials (expanded polystyrene, expanded polyurethane and glass wool). Since the thermal conductivity of porous materials is mainly contributed to by the gas and solid phases, the thermal conductivity can be reduced by increasing the Knudsen effect and improving phonon scattering [134]. Cellulosic porous materials with light weight, high mechanical strength, flame retardance, and heat insulation are expected to replace commercial fossil energy materials for indoor insulation in the future. In the long run, due to the large amount of insulation materials, it is necessary to design cellulose-based thermally insulating materials that can be prepared with low energy consumption on a large scale. The research progress of porous materials based on nanocellulose in heat insulation in recent years has been reported previously in some reviews [24].

**Figure 6:**
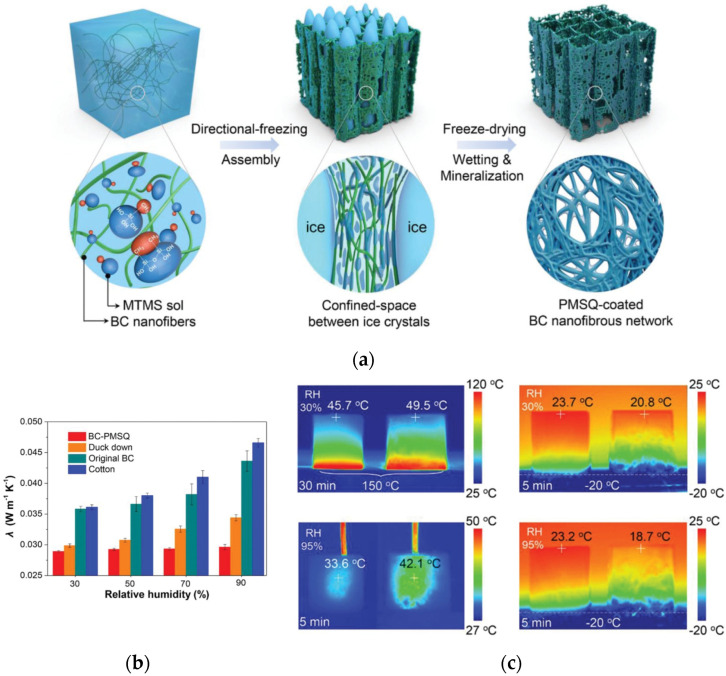
Nanoellulosic hybrid aerogels for thermal insulation. (**a**) Processing principles and synthesis of the BC–PMSQ hybrid aerogels. (**b**) Thermal conductivities λ of BC–PMSQ, down feathers, and pure BC membranes. (**c**) Durable thermal insulation performance was evaluated by optical and infrared images. Adapted with permission from Ref. [132], Copyright 2021, Wiley-VCH GmbH.

For example, a mixed aerogel with a high mechanical compression ratio (≈99%) and superhydrophobicity (≈168°) by using BC and methyltrimethoxysilane (MTMS) was reported (Figure 6a) [132]. Improving moisture-proof performance to prevent water penetration is the key premise to ensuring the thermal insulation quality of aerogel. The layered porous structure ensures the ultra-light and thermal insulation properties of aerogel. The aerogel has a strong superhydrophobicity to withstand high humidity due to its fibrous nanostructures, hydrophobic surface parts and stable nanofiber framework. When the relative humidity changed from 30% to 90% (Figure 6b), the thermal conductivity of aerogel remained almost constant. Under experimental conditions (−20 to 150 °C), the thermal insulation performance of hybrid aerogel is comparable to that of down (Figure 6c). Therefore, the aerogel may be a good choice for thermal protection under extreme temperature and humidity. It can be formed in a variety of shapes by freeze-forming and can be scaled up to any desired size for future industrial applications.

### 5.3. Optically Responsive Soft Etalon

Stimuli-responsive optical hydrogel provides a broad platform for the development of smart materials and has been integrated into a variety of optical filters, sensors, indicators and diodes. For example, hydrogels exhibit strong color changes when the periodicity of the structure is stimulated externally. However, it is very difficult to prepare photonic crystals of such high quality. A key advantage is that fine chemical modifications of hydrogel precursors can control the mechanical strength and elasticity of the resultant film. In recent years, since the cellulose itself does not absorb visible light and does not scatter light significantly, cellulose-based optical hydrogels are attracting more attention.

Recently, an optically responsive soft etalon based on a double network cellulose hydrogel was reported [135]. The refractive index and thickness of the hydrogel were changed by the humidity because of optical interference within the metal–insulator–metal (MIM) cavity. Further functionalization of these cellulose hydrogels will facilitate the development of sensors that respond to a variety of external stimuli. In addition, the Zhang group reported cellulose hydrogels with deformation-induced anisotropy that exhibited high toughness by reacting with epichlorohydrin (EPI) in LiOH/urea solution [92]. Force-induced optical anisotropy is derived from force-induced structural orientation. This kind of cellulose hydrogel can be designed as an intelligent soft matter force sensor to sense external forces because of its sensitive force-induced optical anisotropy behavior.

### 5.4. Wound Healing Therapy

Ideal materials for wound healing should have non-cytotoxicity, good biocompatibility, a high moisturizing effect, excellent breathability, and be easy to apply to different types of wounds [136]. To promote wound healing, wound dressings are essential to repair the skin and restore skin function. Cellulose-based hydrogels are an effective treatment material to promote wound repair, and they have attracted significant attention in the field of tissue regeneration because of their unique properties to meet the requirements of wound healing materials [137]. For example, the injectable hydrogel is initially a liquid at room temperature with pre-gelling fluidity which can be applied to any defect or cavity with minimal invasion. After experiencing transformation or responding to pH/temperature changes in a short period of time, the hydrogel will be formed in situ, and can quickly cross-link with the tissues around the wound. He et al. developed a series of injectable pH-responsive self-healing hydrogels based on acryloyl-6-aminohexanoic acid (AA) and AA-N-hydroxysuccinimide (AA-NHS), and further proved its great potential as an endoscopic sprayable bioadhesive material to effectively prevent bleeding and promote wound healing in a pig stomach bleeding/wound model [138].

**Figure 7:**
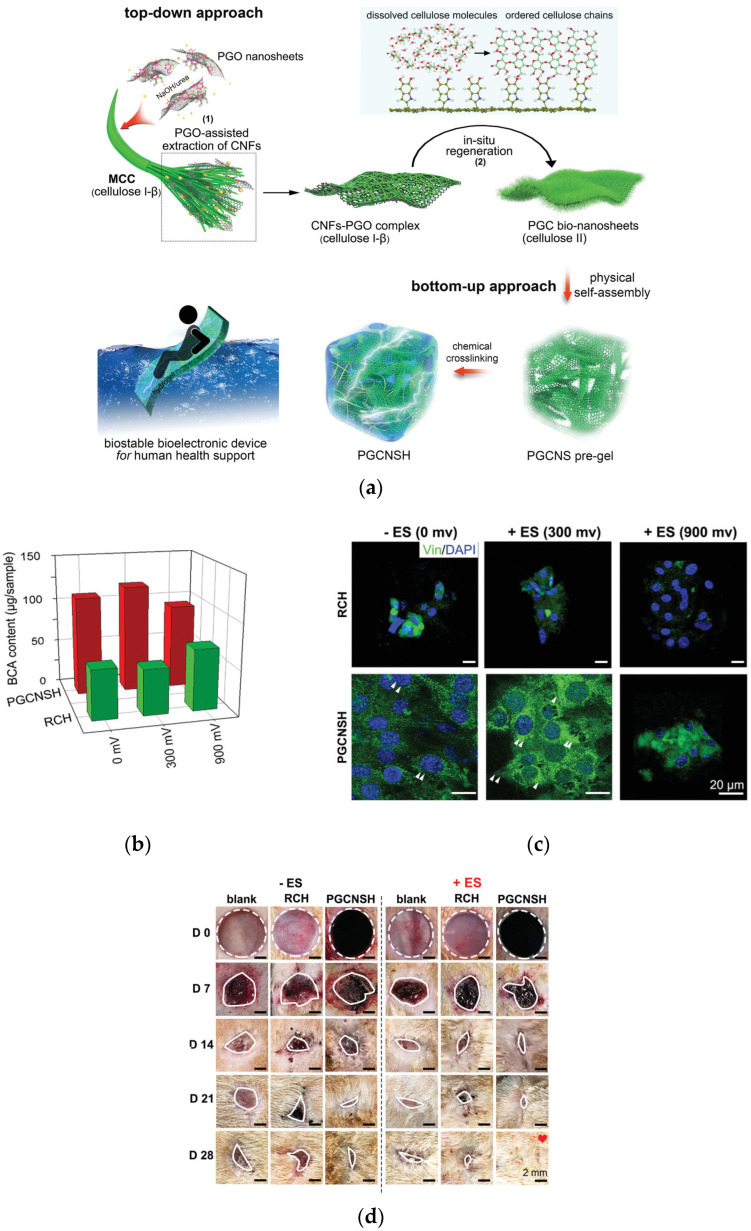
Cellulose-based hydrogels for wound healing. (**a**) Design strategy for 2D conductive cellulose nanosheets and their assembly into biostable and conductive 3D bulk hydrogels. (**b**) BCA content of C2C12 cells on the hydrogels after three days of culturing. (**c**) Immunofluorescent staining for focal adhesion formation of C2C12 cells on day three. Vinculin, a focal adhesion protein, was stained with green, and cell nuclei were counterstained with blue. (**d**) Images of hydrogel-treated wounds with and without electrotherapy. Adapted with permission from Ref. [5], Copyright 2021, Wiley-VCH GmbH.

To further accelerate the wound healing of patients with diabetes, the construction of conductive wound dressings in response to physiological electrical signals and external electric field stimulation at the wound site will help to conduct and distribute electrical signals to the damaged tissue more effectively. Given this, the Lu Group reported a polydopamine-reduced-graphene oxide (PGO)-hybridized cellulose (PGC) bio-nanosheet, and a PGC bio-nanosheet-assembled hydrogel (PGCNSH) with good flexibility, biological stability, electrical conductivity and cell/tissue affinity (Figure 7a) [5]. The effects of hydrogels combined with electric stimulation on cell behavior showed that myoblasts (C2C12 cells) had higher proliferation activity (Figure 7b) and more adhesion spots (Figure 7c) on the PGCNSH hydrogel surface than pure cellulose hydrogel in the absence of electrical stimulation. In addition, the conductive PGCNSH hydrogel can also deliver electrical stimulation in vivo, which can promote the repair of diabetic wounds under the coupling effect of external electrical stimulation (Figure 7d). The results of this study provided an effective synergistic treatment strategy for speeding up diabetic wound repair by coupling electrically conductive cellular-based hydrogel dressings with electrical stimulation. It indicated that conductive PGCNSH hydrogel as an “electronic skin” has the potential to promote chronic wound repair under electrotherapy coupling.

Cook et al. studied a hydrogel dressing which was formed in situ through the reaction between amine-terminated branched poly (ethyleneimine) (PEI) and a bifunctional NHS-activated poly (ethylene glycol) (PEG) cross-linking agent, thiol-ester exchange in the presence of methyl cysteine ( CME ) to leave crosslinking agent and dissolve dressings [139]. This study implements alternative dressings and simple methods for secondary burns.

Human skin is soft and sensitive to environmental changes. Many bionic skin materials have been developed via artificial intelligence, such as wearable sensors and soft robots. These smart devices can convert external stimuli into visual data. However, the complete imitation of human skin’s sensory and sensory characteristics will bring great challenges [140]. The Zhao Group proposed a multifunctional electronic skin based on a HPC, PACA and CNT composite conductive cellulose hydrogel which can not only intuitively feedback external stimuli through color changes, but also through quantitative changes in electrical resistance. The dual-signal sensing capability of conductive cellulose nanocomposite hydrogels is expected to open a new chapter in the design and preparation of multifunctional flexible electronic skin [12].

Moreover, Zhang et al. used a sodium alginate/carboxymethyl cellulose blend hydrogel as biological ink for artificial skin, and confirmed that a SC 4:1 blend hydrogel was most suitable for the 3D printing of artificial skin. This study is of great significance for implantable tissue-engineered skin scaffolds and provides the possibility and basis for the repair of large-area skin defects [137].

### 5.5. Bacterial Infection Therapy

A more stringent requirement for biomedical skin wound dressings is to prevent bacterial infection. Infection is a serious complication of chronic wounds. At present, the treatment of chronic wounds depends on dressings. Such dressings often contain silver as a broad-spectrum antibacterial agent, but inappropriate doses may lead to serious side effects. The alkaline environment is related to chronic wound infection. Due to the release of ammonia and polyamines, the bacterial metabolism may lead to the increase in the local pH value, which may damage the healing of wound tissue and lead to necrosis. Cellulose-based hybrid hydrogels seem to be an effective approach for bacterial infection.

For example, a novel MC—based hydrogel was proposed to release silver nanoparticles (AgNPs) locally through an intelligent mechanism activated by pH changes in infected wounds. By optimizing the physicochemical and rheological properties of MC hydrogel, the optimum process conditions of crosslinker (citric acid) concentration, crosslinker time and temperature can be determined. An ^1^H NMR analysis revealed the role of alkali hydrolysis of ester bonds (i.e., cross-linking bonds) in controlling pH response behavior; the swelling and degradation behavior of MC hydrogels depended on pH and temperature, and it was worth noting that pH triggered the release of AgNPs, which was 10 times higher at pH 12 than at pH 4. MC/AgNPs nanocomposite hydrogels were prepared by in-situ synthesis using MC as a capping and reducing agent. TEM and UV–vis measurements assessed the shape, size, and distribution of AgNPs. Finally, inductively coupled plasma and UV Vis measurements supported the quantitative evaluation of the pH triggered release mechanism of AgNPs to develop systems with enhanced antibacterial activity under alkaline conditions.

In addition, For example, Cai Group proposed a multi-modulus component strategy to prepare a high-strength and high-water content double cross-linked cellulose-Go (DCCG) composite hydrogel [141]. The chemically cross-linked DCCG nanosheet region forms a non-covalent interaction and becomes more elastic and flexible. What’s more, the photothermal conversion performance of Go nanosheets leads to the excellent photothermal antibacterial performance of composite hydrogels. This study is expected to provide new ideas for the construction of high performance and multifunctional composites from natural polymers. Also, Johnson et al. developed a drug-loaded hydrogel composed of CNF and κ-carrageenan oligosaccharide nanoparticles [142]. They chose two antibiotics as therapeutic agents and prepared hydrogels according to the increase in surfactant concentration. The material has been proved to have antibacterial and anti-inflammatory properties and can be used to treat periodontitis.

### 5.6. Drug Delivery

It is essential to control the release of drugs, as the pharmacological purpose cannot be achieved with a rapid release. An “ideal” drug carrier system should deliver precise amounts of drugs at some pre-planned rates to provide the desired level of drugs for treatment.

Hydrogels based on cellulose derivatives have important applications as drug delivery systems (DDS) and function as external stimuli, such as body temperature and variable pH ranges in different parts of the body, to improve the controlled release of drugs. The double-layer hydrogels have great potential to develop into a novel functional sustained drug delivery system [143]. Cellulose-based hydrogels prepared by physical or chemical methods have different structures and swelling degrees. Compared with the hydrogels formed by physical self-association, chemical crosslinking hydrogels can be loaded with greater amounts of drugs that they release faster.

In recent years, in the treatment of oral diseases, the treatment method of directly releasing drugs into the mouth may be used. There is a large amount of saliva flowing through the oral mucosa in the mouth. Due to the special environment of the mouth, hydrogels with long-term adhesion ability are needed to achieve local medication. HPC hydrogel can be used to prepare a bioadhesive hydrogel system by combining HPC with a polyacrylic acid (PAA) lactose non-adhesive backing layer, which can be used to treat aphthous ulcers by releasing triamcinolone acetonide [144]. Traditional insulin injection is carried out by subcutaneous injection, and long-term repeated injections will lead to reduced patient acceptance. In order to avoid these disadvantages, pH-responsive hydrogels prepared by acrylate grafting of CMC and PAA were developed for oral administration of insulin to increase patient comfort [145]. Another example is that the dual functionalized L-Histidine conjugated chitosan-cellulose nanohybrid hydrogel embedded green zinc oxide nanoparticles were formulated as a sustained drug delivery carrier for the polyphenol drugs–Naringenin, Quercetin, and Curcumin [146].

### 5.7. Anti-Tumor Immunotherapy

Thermosensitive hydrogels help to improve the local and remote effects of cancer immunotherapy. Wang mixed surface-modified nanocellulose with hexadecyl amine as a long chain to construct a cellulose hydrogel network. The hydrogel has been successfully applied to the control and targeted delivery of paclitaxel and has achieved a remarkable anti-tumor effect [147]. The hydrogel system is widely used in humans to block the continuous release of cytotoxic T lymphocyte-associated protein-4 from immune checkpoints by nitric oxide donors and antibodies to achieve efficient and durable anti-tumor immunotherapy. Due to its unique hydrogel formation and degradation characteristics, it can maintain the retention of drugs in tumor tissues, which are triggered and released by the tumor microenvironment, and form in situ micelles suitable for lymphatic absorption.

### 5.8. Tissue Engineering

Cellulose-based hydrogels are immensely important for tissue engineering. When hydrogel is used as a scaffold, it can be used in many aspects of tissue engineering, arthroscopy, vascular stents and skin stents. In tissue engineering, tissue function is affected by cell adhesion, proliferation, differentiation and maturation. Therefore, biocompatibility, bioactivity and biomechanics of materials are critical requirements. Biomaterials need the above three points to support tissue regeneration without eliciting any adverse local or systemic reactions in the eventual host.

For example, Guo et al. applied a small amount of epichlorohydrin to slightly pre-crosslink the cellulose chain to form a permeability network to regulate the rheological properties and to form a loose crosslinking point to regulate the self-assembly of the cellulose chain to obtain excellent mechanical properties. Printed cellulose hydrogel has biomimetic NF topology and remarkable tensile and compressive strength (5.22 and 11.80 MPa), and toughness (1.81 and 2.16 MJ/m^3^). The original cellulose hydrogel (ALCOGEL) was prepared by Guo et al. using ethanol as an antisolvent. The mechanical properties of biopolymer materials were improved and adjusted by controlling the fiber arrangement, and both of them are expected to be used in tissue engineering [148].

At present, the main method for the treatment of coronary artery disease is to implant vascular stents and shape memory alloys into the dilated artery. Shi et al. constructed a bi-directional shape memory cellulose scaffold which can be adjusted a by mild solution (such as water and alcohol), has excellent biocompatibility, and can support the left coronary artery or left main coronary artery in an open state [149].

## 6. Conclusions and Future Outlook

In the past few decades, the rapid development of biomedical engineering has brought great opportunities and challenges to cellulose-based hydrogels. On the one hand, cellulose is abundant, renewable, green biodegradable and an eco-friendly building block. On the other hand, cellulose-based hydrogels have become a research hotspot in the field of functional materials because of their outstanding characteristics such as excellent flexibility, stimulus-response, biocompatibility, and degradability. With the increasing and deepening of hydrogel research, cellulose-based hydrogel properties are constantly evolving in an attempt to match the multifunctional polymer materials. For example, multi-functional cellulose hydrogels combine self-healing, high strength, adhesion and conductivity. Therefore, the design and construction of multi-functional cellulose-based hydrogel materials to meet the application requirements of different fields is the focus of future research.

At present, most of the research is focused on functional cellulose-based hydrogels as flexible wearable sensors, drug carriers, and wound dressings. However, some disadvantages need to be solved, such as the easy loss of the sensor signal, poor antifreeze performance in low temperatures, the no-matching chemical force between the sensor and the organization, foreign body reaction, and performance loss. At the same time, most functional cellulose hydrogels are still in the experimental stage and have not been put into industrial production because of technical immaturity. Therefore, a lot of research work is needed to popularize functional cellulose-based hydrogels in daily life, realize their commercialization, and give full play to their application potential. Cellulose-based hydrogels have a long way to go in the future.

## Figures and Tables

**Table 2:** Modification method and classification of cellulose derivatives.

Methods	Classification
Physical modification	film cellulose;
	microcrystalline cellulose;
	spherical cellulose;
	nano-cellulose;
Chemical modification	degradation reactions: acid-base, oxidative, biodegradation, mechanical processing;
	hydroxyl derivative reactions: nucleophilic substitution, graft copolymerization, cross-linking reaction, esterification reaction, etherification reaction.

## Data Availability

The data used to support the review summary of this paper are included within the article.
